# Epidemiology and severity of traumatic dental injuries in permanent teeth: A 20-year retrospective study

**DOI:** 10.1590/0103-6440202305257

**Published:** 2023-07-17

**Authors:** Walbert A. Vieira, Andrea C. Pereira, Jaqueline Lazzari, Vanessa G.A. Pecorari, Brenda P.F.A. Gomes, José Flávio A. de Almeida, Caio C. R. Ferraz, Eduardo C. A. Santos, Júlio Vargas-Neto, Adriana de-Jesus-Soares

**Affiliations:** 1 Department of Restorative Dentistry, Endodontics Division, School of Dentistry of Piracicaba, State University of Campinas, Piracicaba, Brazil.; 2 Paulista University - UNIP, São Paulo, Brazil.; 3 Department of Paediatric Dentistry, Orthodontics Division, School of Dentistry of Piracicaba, State University of Campinas- UNICAMP, Piracicaba, Brazil.

**Keywords:** Tooth injuries, Epidemiology, Risk Factors, Tooth Avulsion

## Abstract

This study aimed to assess the traumatic dental injuries (TDIs) in permanent dentition among patients who attended at the outpatient clinic of a Brazilian dental school, during the last 20 years, and to investigate factors associated with the severity of these injuries. Clinical records of patients who attended a specialized center for dental trauma care in Brazil presenting at least one TDI in a permanent tooth, between the years 2000 and 2019, were reviewed. The data recorded were sex, age, affected arch, etiology, number, and type of the teeth affected, and classification and severity of the TDIs. The diagnosis and classification of the TDIs were based on the guidelines of the International Association of Dental Traumatology (IADT). The severity of each patient's injuries was defined as mild, moderate, or severe. Descriptive statistics, chi-square and multinomial regression analyses were used to evaluate the results. The significance level was set at 5%. A total of 837 clinical records were included, totaling 2357 teeth. Males were more prevalent than females. The patients' age ranged from 5 to 71 years. The most common traumas were avulsion (n=512) and uncomplicated enamel-dentin fracture (n=488). Univariate analyses showed that there was a statistically significant association between age group (p=0.004), etiology (p=0.000) and number of teeth affected (p=0.000) with severity of dental trauma. In conclusion, TDIs that occurred in Piracicaba and region are epidemiologically similar to those found worldwide, and that more severe injuries are related to age range, etiology and number of teeth affected.

## Resumo

Esse estudo objetivou avaliar as injúrias dentárias traumáticas (IDTs) na dentição permanente entre os pacientes que compareceram ao ambulatório de uma faculdade de odontologia brasileira, durante os últimos 20 anos, e investigar fatores associados à severidade dessas lesões. Os registros clínicos dos pacientes que compareceram a um centro especializado de atendimento em traumatismo dentário no Brasil apresentando pelo menos uma IDT em dente permanente, entre os anos de 2000 e 2019, foram revisados. Os dados registrados foram sexo, idade, arco dental afetado, etiologia, número e tipo dos dentes afetados, e classificação e severidade das IDTs. O diagnóstico e a classificação das IDTs foram baseados nas diretrizes da Associação Internacional de Traumatologia Dentária (AITD). A gravidade das lesões de cada paciente foi definida como leve, moderada ou severa. Estatísticas descritivas, teste qui-quadrado e análises de regressão multinomial foram usadas para avaliar os resultados. O nível de significância foi fixado em 5%. Um total de 837 registros clínicos foi incluído, totalizando 2357 dentes. O sexo masculino foi mais prevalente que o feminino. A idade dos pacientes variou de 5 a 71 anos. Os traumas mais comuns foram avulsão (n=512) e fratura não-complicada do esmalte-dentina (n=488). As análises univariadas mostraram que houve associação estatisticamente significativa entre a faixa etária (p=0,004), etiologia (p=0,000) e número de dentes afetados (p=0,000) com a gravidade do traumatismo dentário. Em conclusão, as IDTs que ocorreram em Piracicaba e região são epidemiologicamente semelhantes aos encontrados em todo o mundo, e que lesões mais graves estão relacionadas à faixa etária, etiologia e número de dentes afetados.

## Introduction

Traumatic dental injuries (TDIs) represent a serious public health problem. It is estimated that the prevalence of TDI varied between 18% and 25% in permanent dentition and could occupy the 5^th^ position in the ranking of most common injuries ^1^. In countries with a reduced prevalence of caries and periodontal disease, TDIs have been recognized as one of the main causes of tooth loss ^2^.

 Epidemiological studies conducted in TDIs care centers have shown that boys are more affected than girls, and that maxillary central incisors are the most affected teeth ^3^ ^,^ ^4^. The main etiologies include falls, bicycle accidents, and sports practices ^3^ ^,^ ^4^. Regarding the types of TDIs, the most frequent are enamel fracture, uncomplicated crown fracture, avulsion, and luxation ^3^ ^,^ ^5^.

 The sequelae of TDIs can affect the patient physically, psychologically, or financially, and vary according to the severity of the type of injury ^5^ ^,^ ^6^ ^,^ ^7^. Previous studies have concluded that the prevalence of root resorption is higher in cases of avulsion and luxation than in cases of concussion or subluxation ^8^ ^,^ ^9^ ^,^ ^10^. In addition, patients who suffer a crown fracture concomitant to subluxation are more likely to suffer pulp necrosis than patients who suffer only enamel fracture or subluxation ^11^ ^,^ ^12^. In this scenario, the correct identification and early intervention of the TDIs play a key role in the good prognosis of the case and in the return of the quality of life of patients and their relatives.

 In Brazil, although several epidemiological studies in the area of dental traumatology have been published in recent years, most have only evaluated the prevalence of TDIs in permanent teeth in the general population ^13^, and there are few studies that focus on epidemiology and factors associated with severity of the cases treated in specialized care centers ^7^ ^,^ ^14^ ^,^ ^15^.

 Thus, given the importance of knowing the epidemiological profile and severity of cases of TDIs in the development of preventive measures and planning of appropriate treatments, this study aimed to describe the epidemiological pattern of the TDIs treated at an outpatient clinic of a Brazilian dental school over a period of twenty years and to investigate characteristics associated with the severity of these injuries.

## Material and methods

The study was approved by the local Research Ethics Committee and was reported according to the guidelines of the Strengthening the Reporting of Observational Studies in Epidemiology (STROBE) ^16^.

 This is a retrospective observational study that reviewed clinical records of patients treated between 2000 and 2019 in the outpatient clinic of the Dental Trauma Care Service of the Piracicaba Dental School, State University of Campinas, Brazil. The Piracicaba Dental School is located in the city of Piracicaba (São Paulo - Brazil) and has been a reference clinic for dental trauma care in permanent teeth for 20 years, offering care to a region of approximately twenty-four cities and 1.5 million inhabitants.

 The clinical records of all patients affected by TDI in permanent teeth treated between January 2000 and December 2019, regardless of sex, ethnicity, or social group were included. Inadequate clinical records with incomplete data that did not allow data evaluation were excluded from the study.

 After the selection of the clinical records, a single trained researcher according to the following aspects collected the data: gender, age group, affected arch, dental group, etiology, number of affected teeth, classification, and severity of the TDI. A senior researcher double-checked the extracted data.

Because this care center is located in a higher education institution, the clinical records were filled out by different professionals (undergraduate, graduate, trainee, and professors). The diagnosis of the TDIs was done on the first visit of the patient and was based on the guidelines of the International Association of Dental Traumatology (IADT) ^17^ ^,^ ^18^. During the first visit, the professionals also collected important information to understand the context in which TDI occurred such as how, when, and where the TDI occurred, whether the patient had an emergency management or not, and report of any prior TDI.

The severity of the TDI of each patient was classified as mild, moderate, or severe, according to the affected tissue (dental or periodontal), using a classification based on a previous study ^(^ ^19^ (Box 1). The classification of the severity of the TDIs was based on the diagnosis reported on the clinical records.

**Box 1:**
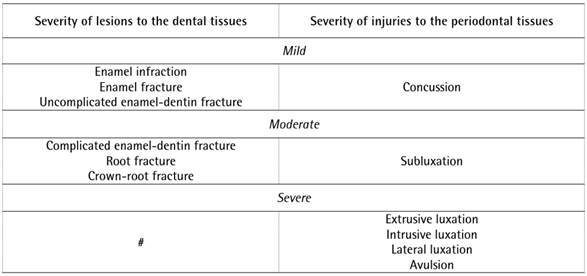
Classification of the severity of the TDIs observed in the study.

# No injury to the hard dental tissue was classified as severe.

To determine the overall severity of patients who had more than one TDI, the most severe lesion was considered, according to the following classification, in descending order: avulsion, lateral luxation, intrusive luxation, extrusive luxation, crown-root fracture, radicular fracture, complicated enamel-dentin fracture, subluxation, concussion, uncomplicated enamel-dentin fracture, enamel fracture, and enamel infraction. For patients with TDIs of the same severity in both dental arches, the arch with the most intense lesion was considered. Conversely, in patients with lesions of the same intensity, in the upper and lower arches, the arch with the highest number of teeth involved was considered.

 A descriptive analysis of the categorized variables was performed, and the frequencies and percentages were presented. The independent variables were defined as: gender (male or female), age range (≤ 14 years, 15 to 25 years, and ≥ 26 years), etiology of TDI (Fall from standing height, bicycle accident, sport, traffic, and others), arch (upper or lower), and the number of teeth involved (1 or 2, and ≥ 3). To assess the association of independent variables with TDIs severity (dependent variable), a univariate analysis was performed using the chi-square test, considering a significance level of 5%. Additionally, a multinomial regression analysis was performed, modeling the variables that showed significance in the chi-square test. Mild severity was used as a reference, and the results of the adjusted model were presented as odds ratios (ORs) followed by the respective confidence intervals (95% CIs). The statistical program used was SPSS 21 (IBM Corporation, Armonk, NY, USA).

## Results

 A total of 910 clinical records were identified, of which twenty-six records were excluded due to incomplete data. Therefore, the final sample included 837 clinical records of patients treated from January 2000 to December 2019, totaling 2,357 teeth involved.

 Males were the most affected, with a ratio of 1.8:1 compared to females. The age of the patients ranged from 5 to 71 years (mean 19.5 ± 12.1 years). The most common etiology was falling from a standing height, followed by bicycle accidents. Most patients were classified as having severe lesions. The maxillary central incisors were the most affected, followed by the maxillary lateral incisors (Table 1).

**Figure 1:**
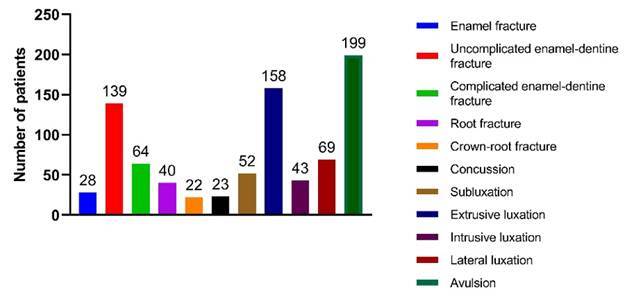
Distribution of the most severe TDIs for each patient.

The most frequent TDI to the dental tissues over the 20 years was uncomplicated enamel-dentin fracture (n= 488) (Table 1), and to the periodontal tissues were dental avulsion (n= 512) and subluxation (n= 460) (Table 1). Considering only the most severe TDI of each patient (n= 837), the most frequent TDIs were avulsion (n= 199 patients), extrusive luxation (n= 158 patients), and uncomplicated enamel-dentin fracture (n = 139 patients) (Figure 1).

Univariate analyses showed that there was a significant statistical association between age (p = 0.004), etiology (p = 0.000), and number of affected teeth (p = 0.000) with the severity of the TDIs (Table 2). For multinomial regression, the etiologies were reclassified according to Table 3.

 The multinomial analysis showed that patients aged 26 years or older (OR - 2.41; 95% CI: 1.36 - 4.27; p = 0.002) and between 15 and 25 years (OR - 1.66; 95% CI: 1.01 - 2.7; p = 0.045) were more likely to have moderate TDI compared to patients aged 14 years or less. Patients who had 3 or more teeth involved were more likely to have suffered moderate and severe TDI than patients who suffered TDI in one or two teeth. Regarding the etiology of trauma, patients who suffered traffic accidents or others (OR - 1.78; 95% CI: 1.13 - 2.79; p = 0.012) or who suffered trauma due to bicycle accidents and during sports practice (OR - 1.51; CI95%: 1.00 - 2.32; p = 0.05) were associated with more severe TDIs than patients who suffered trauma due to falls from standing height. More details of each analysis can be seen in Table 3.

**Table 1:** Clinical and demographic characteristics of the patients included in the study.

	Variables	n	%
Sex	Male	536	64.0
	Female	301	36.0
Age group	≤ 14 y	392	46.8
	15 ~ 25 y	254	30.3
	≥ 26 y	191	22.9
Etiology	Fall from standing height	310	37.0
	Bicycle accident	179	21.4
	Sport	67	8.0
	Traffic	137	16.4
	Others	144	17.2
Arcade	Upper	784	93.7
	Lower	53	6.3
Number of teeth involved	1 or 2	473	56.5
	≥ 3	364	43.5
Type of tooth involved	MCI	1250	53.0
	MLI	561	23.8
	MC	78	3.3
	MPM	30	1.3
	MM	9	0.4
	MdCI	195	8.3
	MdLI	149	6.3
	MdC	44	1.9
	MdPM	29	1.2
	MdM	12	0.5
Affected tissue, per tooth	Dental	554	23.5
	Periodontal	1332	56.5
	Combined	471	20.0
Trauma to dental tissues, per tooth	Enamel infraction	96	8.0
	Enamel fracture	239	21.0
	Uncomplicated enamel-dentine fracture	488	43.0
	Complicated enamel-dentine fracture	161	14.0
	Root fracture	101	9.0
	Crown-root fracture	59	5.0
Trauma to periodontal tissue, per tooth	Concussion	201	10.0
	Subluxation	460	23.0
	Extrusive luxation	433	22.0
	Intrusive luxation	116	6.0
	Lateral luxation	270	13.0
	Avulsion	512	26.0
Severity, per patient	Mild	190	23.6
	Moderate	177	21.9
	Severe	470	54.5

**Table 2:** Association between the severity of the TDI and clinical and demographic characteristics.

Variable		Severity						p value
		Mild		Moderate		Severe		
		n	%	n	%	n	%	
Gender	Male	119	22.2	111	20.7	306	57.1	0.767
	Female	71	23.6	66	21.9	164	54.5	
Age group	≤ 14 y	111	28.3	72	18.4	209	53.3	0.004
	15 ~ 25 y	50	19.7	57	22.4	147	57.9	
	≥ 26 y	29	15.2	48	25.1	114	59.7	
Etiology	Fall from standing height	88	28.4	77	24.8	145	46.8	0.000
	Bicycle accident	36	20.1	35	19.6	108	60.3	
	Sport	19	28.4	11	16.4	37	55.2	
	Traffic	18	13.1	18	13.1	101	73.7	
	Others	29	20.1	36	25.0	79	54.9	
Arch	Upper	182	23.2	167	21.3	435	55.5	0.277
	Lower	8	15.1	10	18.9	35	66.0	
Number of teeth involved	1 or 2	151	31.9	117	24.7	205	43.3	0.000
	≥ 3	39	10.5	60	16.5	265	72.8	

**Table 3:** Multinomial analysis of the association between the severity of the IDTs and age group, number of teeth involved and etiology.

Severity	Variables		p value	OR (95% CI)
Moderate	Age range	≤ 14 y	ref	ref
		15 ~ 25 y	0.045	1.66 (1.012 - 2.723)
		≥ 26 y	0.002	2.41 (1.368 - 4.273)
	Number of teeth involved	1 or 2	ref	ref
		≥ 3	0.011	1.85 (1.152 - 2.983)
	Etiology	Fall from standing height	ref	ref
		Fall by bicycle + Sports	0.790	0.93 (0.564 - 1.545)
		Traffic + Others	0.972	0.99 (0.588 - 1.669)
Severe	Age range	≤ 14 y	ref	ref
		15 ~ 25 y	0.425	1.18 (0.778 - 1.816)
		≥ 26 y	0.071	1.59 (0.962 - 2.639)
	Number of teeth involved	1 or 2	ref	ref
		≥ 3	0.000	4.64 (3.110- 6.931)
	Etiology	Fall from standing height	ref	ref
		Fall by bicycle + Sports	0.050	1.51 (1.00 - 2.321)
		Traffic + Others	0.012	1.78 (1.135 - 2.795)

## Discussion

 This study aimed to evaluate the pattern of TDIs seen in a specialized service of dental trauma care in Brazil and to explore factors associated with the severity of these injuries. The findings showed that tooth avulsion, subluxation, and uncomplicated enamel-dentin fracture are the most common TDIs found over the 20 years period and that the age of the patients, number of teeth involved, and etiology are characteristics associated with moderate or severe injuries.

 Knowledge of the epidemiological profile of patients and injuries treated is important to understand how TDIs are occurring in a given region and thus contribute to the development of preventive measures and planning for care. In the present study, we observed that males were more prevalent than females, which agrees with other similar studies that used samples from patients who attended dental trauma specialized centers ^3^ ^,^ ^4^ ^,^ ^5^. The role of sex as a risk factor for TDIs is extensively discussed in the literature, with some studies indicating a greater predisposition for males ^13^ ^,^ ^20^. Based on the findings of the present study, we can assume that boys have a higher prevalence of TDIs because they culturally exhibit more hyperactive habits and behaviors in daily life than girls, such as the practice of physical contact sports or involvement in dangerous situations ^13^ ^,^ ^20^. However, the statistical analysis showed that sex was not associated with the severity of the TDIs found throughout the study period, which means that both sexes may be prone to suffer the same types of TDIs, despite different frequencies.

 Another important finding of the present study is the higher prevalence of trauma to the maxillary incisors. This result is explained by the vulnerable position of the maxillary anterior teeth in the dental arch, which are the first to receive the direct impact of traumatic forces ^21^. This result agrees with the current literature ^(^ ^3^ ^,^ ^5^ and highlights the importance of a thorough evaluation of these teeth in all episodes of TDIs, even without apparent clinical signs.

 The most severe TDIs found in each patient were tooth avulsion, extrusive luxation, and uncomplicated enamel-dentin fracture. This finding is consistent with what is described in previous epidemiological studies ^3^ ^,^ ^5^ and can be explained by the fact that patients who suffer more severe traumatic injuries or that cause aesthetic damage resort more often to specialized care services, while patients who suffer mild trauma without aesthetic impacts more often neglect the trauma episode. This knowledge is important because the actual epidemiology of dental trauma can be masked by the underreporting of mild cases of TDIs, in which patients do not seek care; in this sense, awareness campaigns are necessary to encourage patients who have suffered any type of TDI to seek specialized care because these injuries can also result in important sequelae ^7^.

The statistical analyses of the present study showed associations between the severity of the TDIs and factors such as age range, etiology, and number of affected teeth. The multinomial regression analyses showed that patients aged between 15 and 25 years and equal to or greater than 26 years were more likely to suffer moderate trauma than patients up to 14 years of age. These data may be explained by the different daily habits that may have caused dental trauma, since adolescent and adult patients may be more vulnerable to risky situations ^22^ ^,^ ^23^. This result agrees with a previous study that shows that maxillofacial fractures due to road traffic accidents increased with age ^24^.

 Other important associations observed in multinomial analyses concern the association between the etiology and severity of trauma. The data showed that patients who suffered trauma due to traffic or bicycle accidents, or during sports practice were associated with more severe trauma than patients who suffered falls from standing heigh. This result is directly related to the traumatic forces produced at the time of the accident since traffic or bicycle accidents are higher energy traumas than falling from standing height ^(^ ^24^ ^,^ ^25^; furthermore, these etiologies are primarily responsible for emergency trauma care in which patients do not present only dental lesions, but also severe maxillofacial traumas ^22^ ^,^ ^24^ ^,^ ^26^.

 Finally, patients included in the present study who had 3 or more traumatized teeth were also associated with more severe injuries when compared to patients with one or two traumatized teeth. These findings can also be justified by the biomechanics of the traumatic episode and the stress distribution of the traumatic energy ^(^ ^21^; in other words, to affect various dental elements, it is expected that the stress produced during the trauma had great energy, which would consequently be associated with more severe trauma ^22^ ^,^ ^26^.

 The main limitation of this study is its retrospective design, which makes it impossible to evaluate the cause-effect relationship between the variables investigated and the severity of dental trauma; furthermore, some patients included in this study sought the care center after a long period from the traumatic episodes, which may introduce a patient recall bias at the time of anamnesis and diagnosis of the TDIs.

On the other hand, this is one of the few studies in the literature to present an epidemiological survey of twenty years, in addition to exploring factors associated with the severity of TDIs. This study also emphasizes the importance of a multidisciplinary and properly trained team to compose a dental trauma service, since a significant portion of patients treated present severe injuries that may be combined with maxillofacial injuries. Moreover, awareness-raising services on prevention and emergency management of new IDT cases should be provided in the community where the service is in order to reduce the prevalence of these injuries.

In conclusion, TDIs occurring in Piracicaba and its region are epidemiologically similar to those found in the literature, and more severe injuries are related to the age group, etiology, and number of affected teeth. Due to the high prevalence and complexity of TDIs, this study emphasizes the need for adequate training for dentists to provide early diagnosis and prevention of sequelae. Moreover, this study provides important information to help in structuring new dental trauma services in other universities and countries.
